# Supplementary material for: Proteostasis in dendritic cells is controlled by the PERK signaling axis independently of ATF4
Source: Life Sci Alliance. 2020 Dec 21;4(2):e202000865. doi: 10.26508/lsa.202000865 (PMC7756897; doi:10.26508/lsa.202000865)
Supplement: Supplementary file 1 [file LSA-2020-00865_TableS1.docx]

| **Series** | **doi** | **reference** | **description** | **class** | **replicate** |
| --- | --- | --- | --- | --- | --- |
| **GSE9810** | 10.1186/gb-2008-9-1-r17 | GSM247587 | CD8alpha cDCs | cDC1 | 1 |
| **GSE9810** | 10.1186/gb-2008-9-1-r17 | GSM247588 | CD8alpha cDCs | cDC1 | 2 |
| **GSE9810** | 10.1186/gb-2008-9-1-r17 | GSM247589 | CD11b cDCs | cDC2 | 1 |
| **GSE9810** | 10.1186/gb-2008-9-1-r17 | GSM247590 | CD11b cDCs | cDC2 | 2 |
| **GSE9810** | 10.1186/gb-2008-9-1-r17 | GSM247591 | pDCs | pDC | 1 |
| **GSE9810** | 10.1186/gb-2008-9-1-r17 | GSM247592 | pDCs | pDC | 2 |
| **GSE9810** | 10.1186/gb-2008-9-1-r17 | GSM247593 | NK cells | NK | 1 |
| **GSE9810** | 10.1186/gb-2008-9-1-r17 | GSM247594 | NK cells | NK | 2 |
| **GSE9810** | 10.1186/gb-2008-9-1-r17 | GSM247595 | B lymphocytes | B | 1 |
| **GSE9810** | 10.1186/gb-2008-9-1-r17 | GSM247596 | B lymphocytes | B | 2 |
| **GSE9810** | 10.1186/gb-2008-9-1-r17 | GSM247597 | B lymphocytes | B | 3 |
| **GSE9810** | 10.1186/gb-2008-9-1-r17 | GSM247598 | CD8 T lymphocytes | T_CD8 | 1 |
| **GSE9810** | 10.1186/gb-2008-9-1-r17 | GSM247599 | CD8 T lymphocytes | T_CD8 | 2 |
| **GSE2389** | 10.1016/j.immuni.2005.01.016 | GSM44979 | CD4 T | T_CD4 | 1 |
| **GSE2389** | 10.1016/j.immuni.2005.01.016 | GSM44982 | CD4 T | T_CD4 | 2 |

Supplementary Table 1: List of data sets used in the GSEA of immune cells
