# Supplementary material for: Proteostasis in dendritic cells is controlled by the PERK signaling axis independently of ATF4
Source: Life Sci Alliance. 2020 Dec 21;4(2):e202000865. doi: 10.26508/lsa.202000865 (PMC7756897; doi:10.26508/lsa.202000865)
Supplement: Supplementary file 2 [file LSA-2020-00865_TableS2.docx]

Supplementary Table 2: List of data sets used for ATF4 and clSR gene signatures in the GSEA.

| **Name** | **Source** | **doi** | **Description** | **Gene_count** |
| --- | --- | --- | --- | --- |
| **Han_ATF4_targets** | Han et al., 2013, Nat. Cell. Biol. | 10.1038/ncb2738 | List of ATF4 and CHOP target genes that have binding peaks within 3kb from TSS of annotated gene.”, Select genes with “Overlap=Common OR ATF4_Only” | 472 |
| **Han_CHOP_targets** | Han et al., 2013, Nat. Cell. Biol. | 10.1038/ncb2738 | List of ATF4 and CHOP target genes that have binding peaks within 3kb from TSS of annotated gene.”, Select genes with “Overlap=Common OR CHOP_Only” | 321 |
| **Han_ATF4_CHOP_Common_targets** | Han et al., 2013, Nat. Cell. Biol. | 10.1038/ncb2738 | List of ATF4 and CHOP target genes that have binding peaks within 3kb from TSS of annotated gene.”, Select genes with “Overlap=Common” | 218 |
| **Guan_ER_Prot_Proc_Pathway** | Guan et al., 2017, Mol. Cell. | 10.1016/j.molcel.2017.11.007 | “ER protein processing pathway (35 genes) identified from Tg:16h vs. Tg:1h genome-wide analysis” | 34 |
| **Guan_Congruent_Up_16h** | Guan et al., 2017, Mol. Cell. | 10.1016/j.molcel.2017.11.007 | Get on request | 546 |
| **Guan_Translation_Up_1h** | Guan et al., 2017, Mol. Cell. | 10.1016/j.molcel.2017.11.007 | Get on request | 1336 |
| **C2_KEGG** | Broad Institute | <https://www.gsea-msigdb.org/gsea/index.jsp> | KEGG Pathway | 186 Pathways |
