# Supplementary material for: Proteostasis in dendritic cells is controlled by the PERK signaling axis independently of ATF4
Source: Life Sci Alliance. 2020 Dec 21;4(2):e202000865. doi: 10.26508/lsa.202000865 (PMC7756897; doi:10.26508/lsa.202000865)
Supplement: Supplementary file 3 [file LSA-2020-00865_TableS3.docx]

| Supplementary Table 3: Genes upregulated only in WT vs ATF4 -/- MEFs upon tunicamycin treatment. | | | | | |
| --- | --- | --- | --- | --- | --- |
| Car6 | 4932422M17Rik | Osbpl2 | Hagh | Dym | Asph |
| Derl3 | Gpt2 | 4930431P03Rik | Dusp18 | Usp31 | Ppib |
| Cox6a2 | Rab38 | Slc5a6 | Erlin1 | Rrbp1 | D6Wsu116e |
| Acox2 | S100a7a | Lims2 | Camk1 | Gm10734 | Gtf2a1 |
| Csn3 | Mtm1 | 2610528E23Rik | 2210008F06Rik | 1810010H24Rik | Cyb5b |
| Slc6a12 | Stbd1 | Klra5 | Kctd4 | Mllt4 | Zfp945 |
| Angptl6 | Slc7a1 | Tmcc3 | Camk1d | Cdv3 | Pusl1 |
| 2310002L13Rik | Ptrh1 | Sft2d2 | Tle1 | Plcxd2 | Ssr4 |
| Chchd10 | Shmt2 | XM_124146 | Uprt | Ahnak | Gpr85 |
| Soat2 | NAP112914-­‐1 | Mapk6 | Serpinb9g | Tmem97 | A630033H20Rik |
| Slc6a9 | Chka | Slc1a5 | Otud1 | Ddr2 | Aup1 |
| Chac1 | Gtpbp2 | Mthfd2 | Pard3 | Mtdh | 2310046K23Rik |
| B4galnt2 | Hoxa1 | Fam195a | Xpot | Eif1a | 4732491K20Rik |
| Odz4 | Areg | Eps8 | 2310058N22Rik | Sertad2 | Mal |
| Cth | Clic5 | Cobl | Arhgap33 | Atp2a2 | Gale |
| Tacr2 | Dhrs9 | Tbpl1 | Coasy | Pard6a | A630012P03Rik |
| Cnfn | 5330431K02Rik | Sh3bgrl2 | Slc43a3 | Nfkbib | Hs1bp3 |
| Avil | Flrt3 | Gpr124 | Svip | Timm8a1 | Kdm6a |
| Cdsn | Ppef1 | Dpysl3 | Lrrc8b | Gtf2h1 | Thyn1 |
| Gm10639 | Gpr137b-­‐ps | Akna | 2700007P21Rik | Rnh1 | Gnat2 |
| Gpr137b | Sh3tc2 | Them4 | Mfsd11 | LOC100044193 | Camk2d |
| Fgf21 | Sgcd | Nars | Bcl2l1 | Gls | Fip1l1 |
| Msln | Mamdc2 | Aqp5 | Tcea1 | Slc26a6 | Gm13238 |
| Tmem179 | Asns | XM_485592 | BC022687 | Slc2a5 | Klk10 |
| Vipr2 | Dennd4a | Slc25a29 | Slc25a37 | Ahi1 | 2210011C24Rik |
| Cyb5r2 | Kcnt2 | Gm12060 | Zfand2a | Lyrm4 | Clcn6 |
| Akr1b7 | Mei4 | Stt3b | Gmppb | Bend6 | Ogt |
| Sh2d6 | Phgdh | Ormdl2 | Calcb | Zbtb7b | Kcnma1 |
| Gm3776 | Aig1 | Mpp6 | Stard5 | Sphk2 | Mrps18b |
| Stc2 | Cpeb1 | Amotl1 | Pck2 | Mtss1 | Fads2 |
| Tmem47 | Renbp | Zfp57 | 1810029B16Rik | Slc35c2 | 4922501C03Rik |
| Gm8709 | Mtap1b | Larp1b | Insig1 | Crebl2 | H2-­‐M10.3 |
| Fibin | Ecel1 | Siah2 | Sh3bp2 | LOC100504608 | Far1 |
| Adm2 | Klrg2 | XM_910588 | Cebpg | Twistnb | Nnmt |
| Nrcam | Gm129 | Atp6v0b | AK086332 | AA467197 | Pppde2 |
| Amz1 | Lck | Cars | B3gnt2 | Tmem60 | Cgrrf1 |
| Slc4a11 | NAP111644-­‐1 | Gsta2 | 9130230N09Rik | Eif3c | Try5 |
| Myom2 | Slc25a33 | Hdac4 | Lrrc16a | Jdp2 | Pacs2 |
| Ttc9 | Pyhin1 | NAP112102-­‐1 | Samd12 | Cpeb2 | Rasal1 |
| Cyb5r1 | Nfxl1 | Atp2a3 | Avpi1 | Lrrfip1 | Ube2j1 |
| Gadd45a | Dusp5 | 1110018J18Rik | Lins | 4932412D23Rik | Eps15 |
| Aldh1l2 | Prkg2 | Gm11595 | Sco2 | 5430411K18Rik | Scpep1 |
| Ndrg1 | Rtn4r | Fez1 | Ak5 | Cds2 | 0610007P14Rik |
| Tmem184a | Ascc2 | Tspan15 | Lamb3 | Plcg2 | 4932425I24Rik |
| Ppp1r15a | Cr1l | Duoxa1 | Slc38a1 | Mdfic | Mtap6 |
| Cyp3a13 | Fads3 | Akap2 | Pde10a | Deb1 | Hax1 |
| Wars | Lonrf3 | Slc38a7 | Slc39a11 | XM_909351 | Ciapin1 |
| Sspo | Mtap2 | BC052688 | Cspg4 | Krtap4-­‐8 | Gcnt1 |
| Atad3a | Ptpn22 | Kprp | Uap1l1 | Uqcrq | D13Ertd608e |
| Fam171b | Abca8b | Rps6ka2 | Entpd5 | Mettl22 | Slc25a38 |
| Ercc1 | Gcat | Ostn | Socs2 | Gm7303 | Cln8 |
| Mfsd2a | Slc33a1 | Tead4 | Crls1 | Lars | NAP060966-­‐1 |
| Gm8096 | NAP114398-­‐1 | Meis2 | Csnk2b | Rdh11 | 4930438A08Rik |
| Tbc1d2 | Rnf39 | Gm2016 | Plagl1 | Hapln4 | Hspa13 |
| Itpr2 | Emp2 | Ank2 | NAP112224-­‐1 | Lphn3 | Lman2 |
| Lpin3 | Rab39b | Gsto1 | Slc35e4 | A330009N23Rik | Cdr2l |
| Pla2g12a | Vat1 | Arl5b | Srprb | Timm10 | Det1 |
| Tigit | 0610010O12Rik | Klra22 | Clptm1l | 0610007L01Rik | Tmem181a |
| 4930506M07Rik | Tmeff2 | Slc20a1 | Srm | Pqlc2 | D030028A08Rik |
| Reep6 | 1700016K19Rik | Recql4 | Ipo7 | Klf8 | Parvb |
| Itgb7 | Slc35f2 | LOC236220 | Als2cl | P2rx3 | Agpat5 |
| Perp | Hmox1 | Ass1 | Hspa9 | Lass6 | Tom1 |
| H2-­‐M10.1 | Gnpnat1 | Pvr | Pecr | Tns3 | 1110067D22Rik |
| Pax8 | Epha2 | Rnf185 | Ece2 | Trio | Slc36a1 |
| Cyp2c44 | Plk3 | Suox | Tymp | Yipf6 | Sec14l1 |
| Klf5 | Spcs3 | Fam110c | Tnfaip2 | Cyld | Trappc8 |
| Slc7a11 | Gm2694 | Phf10 | Lonp1 | Pcca | Zdhhc21 |
| Wfs1 | Esyt2 | Ghitm | Tmem192 | Mbtps2 | Klra6 |
| Psph | Cpox | Tmem202 | Tmem209 | Faf1 | Pvrl1 |
| Sybu | Ngef | Sars | Eef2k | Gm9079 | Grpel2 |
| Plcl2 | Mertk | Stau2 | Atf6 | Serpinb9c | BC048403 |
| Ppm1h | Mid1ip1 | Ccdc47 | Eif5 | Eif4g3 | Jagn1 |
| Acot2 | Strbp | Yrdc | Unc5c | Socs6 | Dnaja3 |
| Gch1 | Gdap1l1 | Eprs | Qdpr | Dgkd | Tm2d3 |
| Ptchd1 | Oxsr1 | Scfd2 | Tmem206 | Zyg11b | Ssfa2 |
| Eif4ebp1 | Hs3st5 | Reep3 | Rbm15b | Fut1 | Imp3 |
| Slc7a3 | Rpp25 | Ttll11 | Lrp8 | LOC100504857 | Fam185a |
| Hmx3 | Paqr3 | Sgsm1 | Snd1 | Gfpt1 | Krtcap2 |
| Nrip2 | Npc1 | Tmem120a | Rwdd4a | 2810432L12Rik | Homer1 |
| Extl1 | Aldh18a1 | Ccnd2 | Mvd | Pkp1 | Cdc42se2 |
| Atf3 | Sqstm1 | Scly | Slc17a9 | Tspyl2 | Lnp |
| Lce1c | Sgms2 | Tmem74 |  |  |  |

This gene expression signature represents the genes that were found up-regulated only in

WT and not in ATF4 -/- MEFs upon a tunicamycin 12h treatment as compared to their untreated counterparts. This list was generated from available public genomic data (GSE49598).
